# An Entropy-Guided Monte Carlo Tree Search Approach for Generating Optimal Container Loading Layouts

**DOI:** 10.3390/e20110866

**Published:** 2018-11-09

**Authors:** Richard Cant, Ayodeji Remi-Omosowon, Caroline Langensiepen, Ahmad Lotfi

**Affiliations:** 1 School of Science and Technology, Nottingham Trent University, Clifton Lane, Nottingham NG11 8NS, UK; 2 Descartes Ltd., Thurmaston LE4 9HA, UK

**Keywords:** optimisation, container loading problem, entropy, monte carlo tree search

## Abstract

In this paper, a novel approach to the container loading problem using a spatial entropy measure to bias a Monte Carlo Tree Search is proposed. The proposed algorithm generates layouts that achieve the goals of both fitting a constrained space and also having “consistency” or neatness that enables forklift truck drivers to apply them easily to real shipping containers loaded from one end. Three algorithms are analysed. The first is a basic Monte Carlo Tree Search, driven only by the principle of minimising the length of container that is occupied. The second is an algorithm that uses the proposed entropy measure to drive an otherwise random process. The third algorithm combines these two principles and produces superior results to either. These algorithms are then compared to a classical deterministic algorithm. It is shown that where the classical algorithm fails, the entropy-driven algorithms are still capable of providing good results in a short computational time.

## 1. Introduction

The Container Loading Problem (CLP) is a well-known *NP*-hard (non-deterministic polynomial-time hard) optimisation problem [1,2,3,4] and has been heavily investigated for more than 30 years [5]. This paper arose from a particular practical case of the problem that occurs in the UK Distribution Centre (UKDC) of NSK Ltd. (Nippon Seiko Kabushiki-gaisha—http://www.nsk.com/), one of the largest bearing suppliers in the world, a manufacturer of bearings globally and the largest in Japan. However, the insights gained are applicable to the general theoretical problem, as well as other practical problem cases.

In NSK’s UKDC, bearings are packed into boxes which are put into cartons and arranged on pallets which are then shrink-wrapped and loaded as individual units onto shipping containers for transportation to customers. A typical pallet weighs 445 kg on average and has to be moved about using forklift trucks. There are four different pallet types/sizes currently used in the UKDC, which makes this a variant of the CLP using weakly heterogeneous rectangular pallets.

Multiple pallets are logically grouped together as a single job, all of which must be shipped together; jobs must not be split between different containers, though a single job may need multiple containers. In previous published work [6], a framework for the selection of pallets from a larger set of pallets to be loaded into each available container was presented. As the cost of the entire cargo in a fully loaded container can range from £24,000 to £1,200,000, it is important to arrange the goods carefully in the container in a manner that allows quick and easy loading and unloading by forklift trucks, to speed up the throughput within the distribution centre, and to minimise the possibility of damage to the goods that could occur from improper packing, which would lead to an increase in costs to the company, required as payment for fines by customers and for the replacement of damaged goods.

Within this paper, a holistic approach to the loading process has been taken to combine simplicity for the forklift drivers with efficiency of space utilisation within the container. This reduces the time that a container spends being loaded, and the number of containers needed for a given single job. In previous work [7], a measure of order and consistency for a layout based on entropy was defined, and it was shown that forklift drivers found low entropy solutions easier for them to understand and achieve. This paper demonstrates how low entropy solutions to the CLP can be efficiently produced by the new technique of using entropy to direct a Monte Carlo Tree Search (MCTS) process [8]. The techniques are also compared with a non-entropy-driven MCTS and with a deterministic algorithm. It shows that the use of entropy in combination with MCTS is the most effective in achieving high fill capacities.

The rest of the paper is organised as follows: Section 2 discusses existing work on the CLP followed by the description of a specific problem that we have addressed here and introduces our new approach for generating layouts using the entropy measure with MCTS in Section 3. Section 4 describes the entropy concept in detail followed by the proposed algorithm in Section 5. Section 6 describes the setup for the experiments performed, and Section 7 shows how the algorithm is used to derive layouts in a variety of cases and assesses the performance of the algorithm. Finally, pertinent conclusions are drawn in Section 8.

## 2. Related Works

In a comprehensive survey paper [5] published in 2013, Bortfeldt and Wäscher found 163 published papers from 1980 to 2011 that dealt directly with the CLP. They showed that interest in this area was increasing, so that nearly a quarter of the papers had been published in the final two years of that period. They concluded that few of the papers to that date dealt with the practical constraints of complete shipment or loading priority, though load stability was often considered. Heuristic methods were well advanced, though few exact and approximation algorithms had been put forward. Though they argued that test problem sets were not available to include practical constraints, many papers in that period make use of the problem set by Bischoff and Ratcliff [9], which had an impact in its consideration of practical constraints that might impact the methods used for solving CLPs.

The two most common heuristic methods for the CLP are the layering and wall-building approach. The layering approach, as seen in [10,11,12], is based on the concept of packing items in a loading configuration from the ground up in layers. The wall-building approach, first proposed in [13], with a variant introduced in [14], is based on filling the container with walls where the walls are rectangular blocks made up of boxes whose depth is determined by the first box placed in them. Eley [15] also presents an approach based on wall-building that builds blocks of identical oriented boxes using a greedy heuristic. Another heuristic approach provided in literature is the AND/OR-graph approach proposed by Morabito and Arenalest [16] in which boxes are represented as nodes in a graph and a cut performed on a box is represented as an AND operation. A sequence of cuts is performed until all nodes found are final. The set of all the nodes and AND operators is the AND/OR-graph. These approaches form the foundation for many heuristic frameworks used for solving the CLP in literature.

Some of the techniques used to attack the CLP included genetic algorithms [11,12,17,18], simulated annealing [19], tabu search [20,21], ant colony optimisation [22,23] and the caving degree approach [24]. These approaches have been explored extensively in the literature, and are often hybridised to solve specific variants of the CLP. More recently, parallel versions of some meta-heuristic approaches have been investigated; an example can be seen in [21]. Taking the real-world problems further, a number of papers combined the problems of container loading with multi-destination deliveries, as collected by Iori and Martello [25]. Although Iori [26] considered loading based on a “last in, first out” principle, the emphasis was on the decisions associated with the choice of vehicles and the travelling salesman route issue, rather than the container layout. More recently, Moura and Bortfeldt [27] discussed how the process could be optimised for distribution to multiple customers with trucks packed in two layers, optimising numbers of trucks used, while Alonso et al. [28] took a mathematical approach to optimise loading and unloading effort by minimising the number of trucks to be used.

After the significant success of combining tree-based searching with Monte Carlo evaluation in the challenging board game, Go [29], the potential for its wider impact was immediately seen and a survey by Browne et al. [30] has cited more than 240 papers using the Monte Carlo Tree Search (MCTS) technique in a range of areas and variants from computer Go through crossword puzzle generation to printer scheduling.

Several previous attempts at the container loading problem have also used this technique. Moura and Oliveira [31] have used MCTS in their work on combining the travelling salesman problem with container loading, but they provided their own Greedy Randomised Adaptive Search Procedure (GRASP) for the CLP aspects of the problem, using the MCTS to direct the combination of load selection and route. Moura and Bortfeldt also used a tree search algorithm for filling trucks in the work mentioned earlier [27] but in this case, the tree search was not Monte Carlo—it was a recursive process to ensure the pallets were stacked in an order suitable for delivery. Edelkamp, Gath and Rohde in [32] also used a sub-variant of MCTS called Nested Rollout Policy Adaptation (NRPA) which is a variant of Nested Monte Carlo Search (NMCS) for both two- and three-dimensional variants of the container loading problem and reported some success.

## 3. Problem Definition and New Approach

In order to address the CLP within the context of the distribution centre, a framework was developed for selecting the pallets to be included in a particular container. The overall framework was devised as a three-stage process: (a) selecting the pallets that could be packed into a container to maximise its weight utilisation, (b) forming stacks of those pallets, and (c) laying out the selected stacks onto the container floor. The first two stages of this process were described in detail in an earlier publication [6]. The present work concentrates on the final step, in which the layout of the pallets within the container is determined.

After completing the first two steps, one is left with the two-dimensional problem of fitting a set of pallet stacks onto the floor of a container. There are four types of pallets and a typical full load would be approximately 40 stacks. The problem can thus be defined as the weakly heterogeneous two-dimensional container loading problem. In practice, this appears to be the most important variant of the container loading problem for the following reasons. Firstly, issues relating to stackability and the loading process make a fully three-dimensional approach unfeasible. Secondly, the strongly heterogeneous version of the problem is usually associated with a scenario in which the goods need to be unloaded in different locations, meaning that the container loading problem has to be combined with a travelling salesman problem as in [25,26,27,28].

When looking for suitable layouts, there are two criteria that need to be satisfied:

1. The layout must fit in the container,
2. The layout must be easy for the forklift drivers to load.

In practice, the second requirement means that the layout should be easy to remember, which means that it cannot be too complicated in structure and should form a regular pattern. As will be seen later, the two requirements mentioned above are well correlated.

Taking influences from the concept of entropy in the domains of physics and information into the domain of graphical scene layouts for computer games, a new interpretation of the concept of entropy has been derived [33,34]. This new measure has been applied to layouts generated by earlier algorithms for the CLP, to provide a quantitative assessment of the ease of loading any particular layout for the forklift drivers. This was explored in [7] where it was found that the measure appeared to correlate with what the forklift drivers considered to be easy layouts to load. Entropy is thus a good candidate as a measure of how well the second requirement has been met.

To make use of this new criterion, it needs to be incorporated into an optimisation algorithm and there are two ways in which this can be achieved. The measure could be used as an overall target, as in the fitness function of a genetic algorithm, or as a local heuristic to guide the process at intermediate stages.

In previous work [35] that directly addressed the first requirement, entropy was used in combination with a length measure as a fitness function in a genetic algorithm. This was deemed unsuccessful and impractical as a large number of layouts (numbering in thousands) had to be generated in order for good results to be produced. This was due in part to the method of operation of the genetic algorithm in question, which at first generated a lot of packed layouts randomly, and then used the combined entropy and length measure as the fitness function with which to select the fittest layouts. A more direct approach was required, using entropy during the initial layout generation process.

After further investigation, it was observed that Monte Carlo Tree search would be a promising framework to use as it has recently been applied with great success in problems that were previously thought of as extremely difficult. For example, tree-based searching and Monte Carlo evaluation are combined and applied to the challenging board game “Go” [29].

Monte Carlo Tree Search methods, as developed to address the large search space and lack of a good evaluation function for the Game “Go”, are characterised as follows:

1. The outcome or evaluation of the search process is carried out only at terminating nodes. This contrasts with more traditional mini-max search methods where an evaluation function is invoked after a relatively limited number of steps.
2. To allow the terminating nodes to be reached there is no attempt to be very thorough in the breadth of search. Instead, a cheap mechanism of “rollout” is employed to reach a true terminating node.
3. Whereas traditional search techniques often rely on “tree pruning” to reduce search breadth, Monte Carlo Tree Search techniques choose the branches to be followed at random and rely on a statistical evaluation of a number of different roll-outs to obtain information about the likely outcome of a step.

During a Monte Carlo Tree Search, at any given step, the following options exist:

Op 1 Choose the next step completely at random.
Op 2 Choose the next step based on a heuristic that can be evaluated directly from the current position.
Op 3 Choose the next step based on statistical information about outcomes that have resulted from earlier roll-outs from the current position. This creates a bias towards choices that have had good results in the past.
Op 4 Modify the outcome of step 3 based on the upper confidence bound. This creates a bias towards choices that have previously been unexplored or little explored.

Note that some kind of weighting between all of these criteria is possible.

The novel contribution of the present work is the introduction of a specific heuristic, the entropy heuristic, which can be used for the second option, Op 2, within the overall Monte Carlo Tree Search scheme.

## 4. Entropy Applied to Container Layouts

The concept of entropy was originally developed in physics and later adapted by Shannon in the context of information theory. It is usually explained at an elementary level as a measure of disorder [36]. We have developed a definition of entropy which emphasises this latter aspect. A version was defined to match well with the instinctive expectations of human operatives and this was successful as verified by experiments reported in [7]. Entropy is defined for Ω equiprobable microstates as:

(1) $$S=k_{B}\ln\Omega$$

where $k_{B}$ is the Boltzmann constant, a physical constant that relates the average kinetic energy of particles in a gas with the temperature of the gas. This is a common model for complex physical systems. For systems in which the microstates have different probabilities, the Shannon information entropy is:

(2) $$S=-\sum_{i}p_{i}\ln\left(p_{i}\right)$$

where *i* is the possible states of the system and $p_{i}$ is the probability of a particular state occurring. The Boltzmann constant in information theory is 1 and is therefore dropped. Hence Shannon entropy relates to the probability distribution that generates the states rather than to the individual states themselves. Note that if all states are equally probable, and there are *N* possible states then the entropy simply becomes:

(3) $$S=N\times \left(-\frac{1}{N}log\left(\frac{1}{N}\right)\right)=logN$$

reducing to the physics definition. In this case, the role of the thermodynamic variables is played by the probability distribution. In order to define the entropy of a configuration of pallets, two tasks must be carried out:

1. Deduce the rule (probability distribution) that generated the configuration.
2. Examine all the alternative arrangements that could be generated by the same rule and assign to each a probability. This would enable the use of Shannon’s formula to generate an entropy value.

In practice, there seems to be no easy way of assigning different probabilities to the different states so equal probabilities are assigned to each. The task is then simply to count the number of ways in which an equivalent layout could be produced. The meaning of the word “equivalent” is to some extent a matter of human judgement. As a simple example of this, consider a sequence of playing cards. If one is not restricted to a single deck and selects two cards in succession, the second card could be identical to the first, and there is only one way to do that. For example, the 4 of spades would be followed by another 4 of spades. The number of equivalent states is just one and the entropy is zero. If the second card has the same number and colour but a different suit, then there are now two options (i.e., 4 of spades followed by 4 of clubs, or a 4 of spades followed by another 4 of spades) and hence the entropy will be log2. If the colour is also different (e.g., 4 of spades followed by 4 of diamonds) then there are now four choices and the entropy will be log4. If the 4 of spades is followed by the 9 of spades, there are 13 options (i.e., any of the 13 spades) and hence the entropy will be log13.

One can separate the entropy associated with the suit from the entropy associated with the number. If the 4 of spades is followed by the 9 of clubs, then there are two suit choices and 13 number choices so the number of options altogether is 2×13=26 and the entropy is log26 or log2+log13. In other words, the entropies associated with different aspects of the arrangement can simply be added. Using this principle the entropy of a longer sequence of cards can be deduced as the sum of the entropies associated with the individual steps.

Note that there are potential ambiguities associated with the deduction of these rules and the resulting entropies. For example, given the sequence 4 followed by 5, one could choose the number entropy to be log13 or one could note the sequential nature and choose the number entropy to be log2. This would be on the basis of the two choices 4→4 or 4→5. Ultimately one should make sure that whatever convention is adopted is used consistently and reflects the requirements of the particular problem at hand. In the following sections, the conventions that have been adopted for the container loading problem will be identified.

For a given arrangement of stacks, the entropy arising from the relationships between the stacks must be calculated. Unlike the sequence of cards discussed above, one cannot be certain which pairs of stacks should be associated together so it is necessary to build a minimum spanning tree that gives the lowest possible total entropy while connecting all the stacks together. We call this the entropy tree. It is computed for each layout when determining its entropy. It should not be confused with the MCTS, which is a higher-level construct. In practice, when the algorithm is running, stacks will be added one at a time and so this tree can be built by adding each new stack at the point that generates the lowest entropy.

The individual stack-to-stack entropies will each be constructed of two components: Selection Entropy and Geometric Entropy. Geometric Entropy can itself be further decomposed into Positional and Rotational Entropies.

### 4.1. Selection Entropy

**Figure 1:**
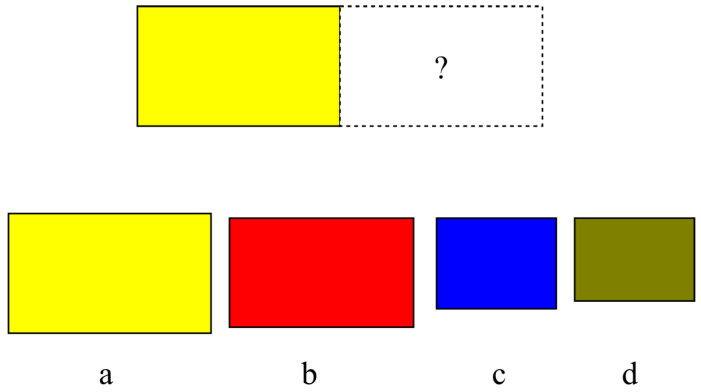
Selection Entropy: if stack type *a* is selected, the entropy contribution is log 1, whereas for *b*, *c*, and *d*, the entropy contribution is due to the number of available stack types (a maximum of log 4 for this problem).

Selection entropy relates to the choices of the types of adjacent stacks. If two similar stacks are placed together then the number of possible choices for doing this is either the number of stacks of this type that remain unplaced in the set, or just one. If each instance of the type of stack is regarded as a different option then the first convention is appropriate. On the other hand, if the stacks are considered truly indistinguishable then the second choice is correct. Likewise, when two different types of stacks are placed together, one can use the total number of stacks in the two sets, or the total number of stacks altogether, or simply the number of different types of stacks. An example problem illustrating this can be seen in Figure 1.

The effects of these choices are quite subtle since the system will always favour placing similar items next to each other regardless of the convention used. In practice, the number of possible choices of placements for stacks of a similar type has been set to one, and that for stacks of different types set to the number of different types of stacks.

These choices for this problem have yielded good results, both in terms of the operator assessment of layouts [7] and the effectiveness of the algorithm presented here. The presence of the Monte Carlo element in the algorithm ensures that the impact of these choices is limited. They cannot prevent a successful configuration from being found, as could be the case if the entropy was used purely deterministically.

### 4.2. Orientation (Rotation) Entropy

**Figure 2:**
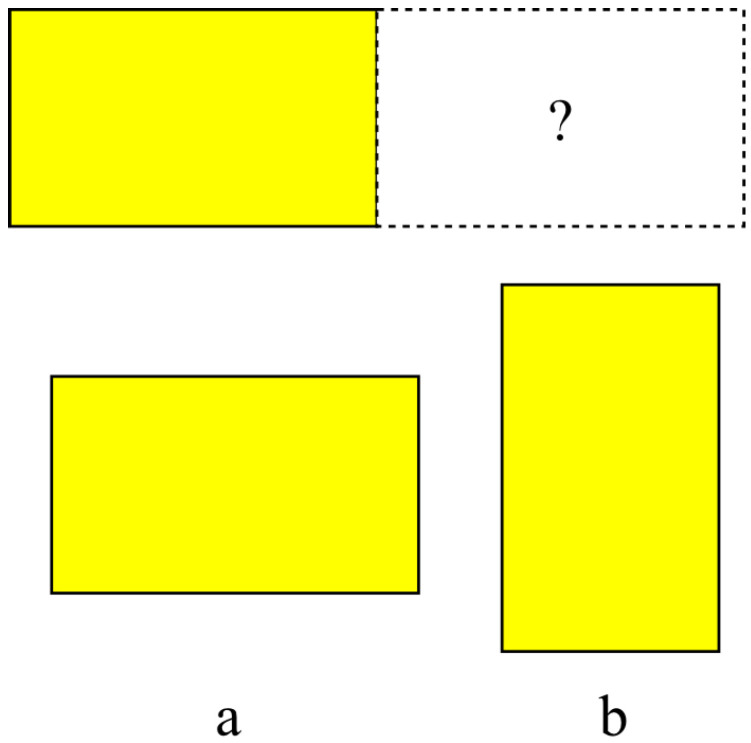
Orientation Entropy: if orientation *a* is selected, then the entropy contribution is log 1, whereas if orientation *b* is selected, the contribution is log 2.

Orientation entropy calculates the entropy due to the relative orientations of adjacent stacks. For any two stacks, if the orientations are the same, the rotational entropy value is set to zero, i.e., log1; otherwise if the orientations are different, the value is set to the log2 (see Figure 2), because only the relative orientations of $0^{\circ }$ and $90^{\circ }$ generate visibly different states. Thus a lower entropy is produced if two adjacent stacks are in the same orientation. Throughout, it is assumed that the stacks are packed parallel to the container walls.

### 4.3. Positional Entropy

Positional entropy is calculated based on the relative positions of items in the plane of the container floor. The relative *x* and *y* coordinates of the centre of the new stack to the centre of the last placed stack are used to determine the entropy contribution from the new stack. In calculating the entropy of two stacks next to each other the following need to be taken into account:

1. The two stacks cannot be placed coincident with each other—they must be displaced by at least half the sum of their dimensions in either *x* or *y*.
2. They can be exactly aligned in the other direction.
3. There is no way of knowing, a priori, which direction has the displacement and which does not.

**Figure 3:**
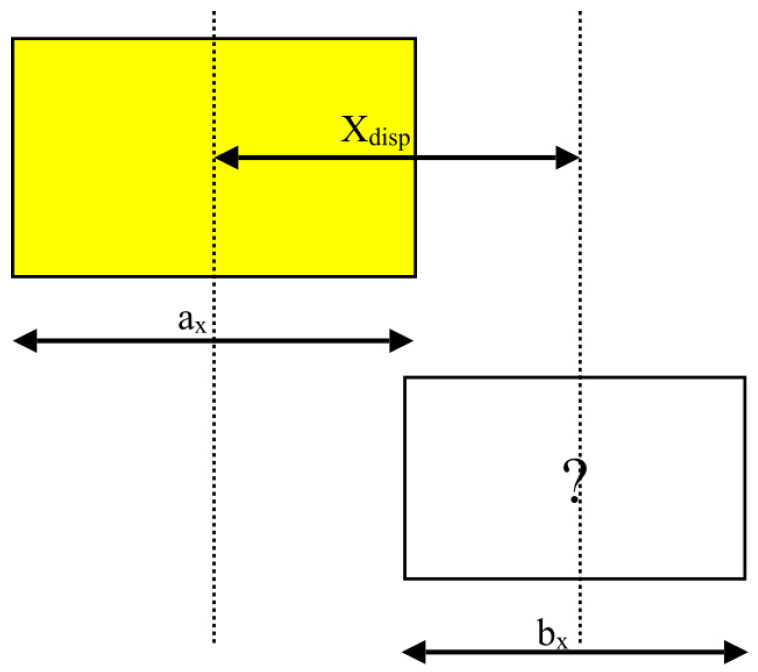
Positional Entropy: this depends on the separation of the centres and the sizes of the stacks in both *x* and *y* directions.

Given two stacks *a* and *b*, define $X_{disp}$ to be the difference in the position of their centres in the *x* direction and $a_{x}$ and $b_{x}$ to be the dimensions of the two stacks in *x* (illustrated in Figure 3). Then the positional entropy in the *x* direction is given by:

(4) $$S_{x}\left(a,b\right)=\ln\left(1+\frac{2X_{disp}}{a_{x}+b_{x}}\right)$$

when

(5) $$X_{disp}\leq \frac{\left(a_{x}+b_{x}\right)}{2}$$

otherwise

(6) $$S_{x}\left(a,b\right)=\ln\left(1+\frac{2X_{disp}}{b_{x}}-\frac{a_{x}}{b_{x}}\right)$$

note that, at the transition, when

(7) $$X_{disp}=\frac{\left(a_{x}+b_{x}\right)}{2}$$

both of these definitions give the same result.

There will be similar equations to define the positional entropy in the *y* direction and the total positional entropy will be the sum of the positional entropies in both directions. Work with experienced forklift loaders showed that they found layouts that had the lowest entropies mirrored and sometimes improved on the layouts they would have generated themselves. The improvements related to both orderliness and compactness in space usage. This indicated that the entropy measure was an appropriate driver for the layout stage of the process.

## 5. Proposed Algorithm

In this section, the placement algorithm is described in detail. Three algorithm variants are analysed. The first is a basic MCTS, driven only by the principle of minimising the length of container that is occupied. The second is an algorithm that uses the proposed entropy measure to drive an otherwise random process. The third algorithm combines these two principles and produces superior results to either. In all of the algorithm variants, the mechanism for placing a new stack follows the pattern described in the following subsection. This guarantees that it will always be possible to create the layout in question in practice using forklift trucks.

**Figure 4:**
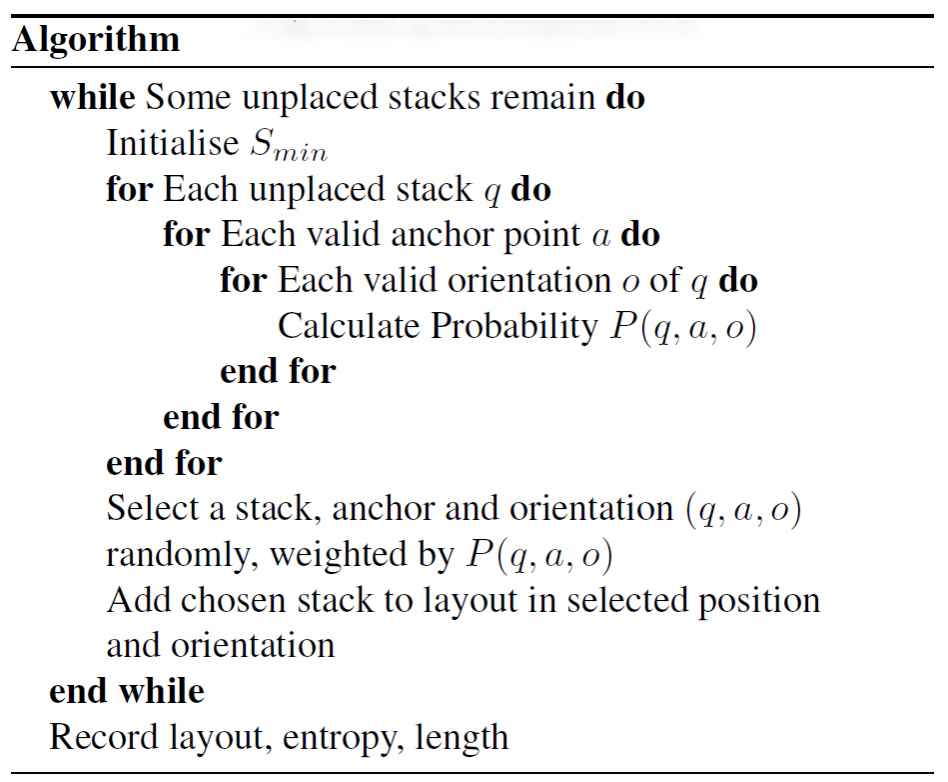
Pallet Stack Placement Monte Carlo Tree Search algorithm.

All of the algorithm variants that have been tested use the structure described by the algorithm shown in Figure 4. The basic principle of all the algorithms is to do a depth-first search. Each attempt to create a layout is taken all the way to the end before a new attempt is made starting from scratch.

In the Entropy-Guided variant, the entropy heuristic is used to bias the choice at each step. In the Length-Driven variant the length of the resulting layout is calculated for each pass and this information is back-propagated up the sequence of placements. Over multiple passes, this results in a tree structure. At each node, the best (shortest) length that has been achieved by any sequence below it is recorded. When a node has not yet been visited the choice of placement is made at random. Later it is biased towards those choices that have resulted in a better result on previous passes.

The Combined Algorithm replaces the purely random choices in the Length-Driven variant with minimum entropy ones. Where the choice was made based on previous success in the Length-Driven algorithm, a combination of entropy and previous success is used in the Combined Algorithm.

### 5.1. Placement Method

In what follows it is assumed that loading is to be performed from one end of the container, the other end being permanently closed. The closed end of the container is referred to as the top and the open end as the bottom. The words “above” and “below” are used consistently with this convention.

The placement of each potential stack within the container is performed by identifying the possible “anchor points”. These are the points that lie on the left inside corners and are accessible from the free space at the bottom of the container. A left inside corner is defined to be a point that has either an existing stack or the container wall to its immediate left and above. In order to trial place a particular stack, a check is made to determine if it will fit and can be moved into position from the container bottom.

### 5.2. Entropy-Guided Algorithm

In our process, as expressed in the pseudocode algorithm shown in Figure 4, each unplaced stack is trial placed in each possible position and valid orientation, and the entropy is calculated. During this process, the minimum entropy $S_{min}$ is also tracked. Equation (8) is then applied to compute the probability P(q,a,o), which can be used to bias the random choice towards lower entropy configurations:

(8) $$P\left(q,a,o\right)=\frac{1}{(1+\omega \left(S\left(q,a,o\right)-S_{min}-1\right)}$$

where ω is a weighting parameter that determines how strongly the entropy affects the outcome. There are two special cases. When ω=0 all the probabilities will be equal and the choice will be completely random. When ω=1 Equation (8) is singular for $S\left(q,a,o\right)=S_{min}$. However, in that case, the lowest entropy choice can be used directly. Occasionally there will be more than one configuration with the same lowest entropy, so a random selection will still be required between these options only.

The Entropy-Guided MCTS needs only local values to decide its path i.e., the entropy associated with adding a particular stack, and so has no need to perform the back-propagation stage used in the other variants. This means that the algorithm is scaleable to allow highly parallel processing without communication, or very long runs without memory issues.

### 5.3. Length-Driven Algorithm

The Length-Driven Algorithm works in exactly the same way as the Entropy-Guided Algorithm except that the probability is calculated using Equation (9).

(9) $$P\left(q,a,o\right)=\frac{1}{(1+\omega \left(L_{max}-L\left(q,a,o\right)-1\right)}$$

The length measure used is the spare length available within a double length container. Larger values therefore represent better results. In this equation L(q,a,o) is the best length so far achieved from the combination (q,a,o) while $L_{max}$ is the best length so far achieved by any choice of (q,a,o) at this level. For unexplored choices within partially explored nodes, the result of Equation (9) is replaced by a fixed probability approximately equal to the probability assigned to the best result so far. For unexplored nodes, a random choice is made. In order to provide the information necessary to implement this equation, the best length information is back-propagated up the tree after each pass.

The Length-Driven Algorithm is inspired by existing MCTS techniques including the one used in [32] for the container loading problem. However, it should be noted that in that paper the test set used for the two-dimensional case was strongly heterogeneous. Techniques such as NMCS and NRPA focus their attention on decisions that are made early in the loading process. However, for the weakly heterogeneous problem here, it is clear that decisions made quite deep into the sequence can be critical. This comes from the observation that the early parts of most successful layouts are repetitive. Therefore an algorithm is proposed that does not explicitly treat the early parts of the sequence any differently. With such a uniform algorithm it is, of course, possible to simulate the behaviour of a non-uniform one by varying parameter values according to the depth within the tree and/or the number of times a node has been visited. However such mechanisms do not appear to achieve any improvement.

### 5.4. Combined Algorithm

For unexplored nodes, the Entropy-Guided algorithm with ω=1.0 is used. Thus all roll-outs are minimum entropy roll-outs. Results are back-propagated as in the Length-Driven Algorithm and thus for fully or partially explored nodes there is a choice between an entropy-guided or length-driven choice. This issue is resolved by using a weighted probability, derived from normalised versions of the outputs of Equations (8) and (9). Combining both entropy and length probabilities for a given stack, anchor point and orientation from Equations (8) and (9) gives:

(10) PC(q,a,o)=αPE(q,a,o)+(1−α)PL(q,a,o)

where both PE and PL are normalised. The possibilities of different choices for this weighting parameter α have been explored and they are presented in the results section.

The values of ω used in Equations (8) and (9) were fixed, based on the best values determined when the algorithms were tested individually.

## 6. Experiments

The algorithms proposed in Section 5 were used in a series of experiments to assess their performance in achieving layouts that optimised space usage while being humanly loadable. The experiments were carried out on AMD A8 four-core desktop machines with 8GB RAM, running Windows 10 with no other user programs active. Each machine was running three instances of the experiment, each of which was explicitly bound to one of the cores. The software was written in C# and no other explicit optimisation or parallelisation was applied.

In order to assess the consistency and robustness of the algorithms, they were applied to a range of situations. The container layouts were categorised in terms of the “fill level”. This denoted the relative proportion of the floor area of the selected stacks compared to that of the container—in other words, a 2-D liquid measure that takes no account of individual stack geometry, only absolute floor area covered. At the UKDC, four different pallet dimensions are used, and so each stack would have one of those four as its base. For each of the different fill levels to be tested in the experiments, fifty sets of stacks were randomly generated, using the 4 possible pallet sizes. Because the largest of the stacks corresponds to about 3% of the container area it is not possible to match a particular fill level exactly. Therefore the notional fill level quoted for each combination is actually a minimum value. Consequently, the largest nominal fill level used was 96%, as larger values could result in an actual fill level exceeding 100%. The use of the notional fill level and the randomisation of the stacks used in each experiment meant that it was quite likely that for high fill levels it might be impossible to get all the stacks into the container once the actual dimensions were taken into account by the algorithm. Since the container loading problem is *NP*-hard there is no practical way of knowing for certain that this is the case for a particular set of stacks.

The algorithms were then applied to produce layouts for each of the random sets of stacks at each of the fill levels. Given the time taken for the physical process of loading using a forklift truck, there is clearly no need for a result in a few seconds; but equally, users cannot afford to wait several hours. A limit of ten minutes was therefore used for the tests as it is a good reflection of user needs. To save time during the tests, runs were terminated as soon as a successful layout was found. In practical usage, it would be desirable to allow the run to continue to produce new lower entropy layouts that might be easier to load. To be usable in a warehouse environment the algorithm must produce its result within a reasonable time.

To give an external comparison, the performance of a deterministic packer, the Skyline algorithm [37] was also evaluated. This algorithm is deterministic but highly dependent on the input ordering. In order to show the best results that this algorithm could achieve, the input files were presorted to group all the similar sized stacks together, ordered from the largest. This algorithm was chosen because its dependence on the input order would allow this simple presorting process to force an ordered layout. This would provide a direct comparison to the entropy-driven method, not just in the ability to successfully fill the container but also in terms of the quality of the configuration that is produced. The presorting process has quite similar effects to our entropy heuristic but lacks the flexibility required to handle the most difficult situations.

## 7. Results and Analysis

The experimental results are presented in two separate sections highlighting the statistical and visual results.

### 7.1. Statistical Results

The overall set of experiments used 50 randomly chosen sets of stacks, for each of 17 different fill levels (73, 75, 77, 79, 80, 81, 83, 85, 87, 89, 90, 91, 92, 93, 94, 95 and 96%). For each algorithm, a variety of different parameter values were tested to determine the most suitable choice for practical use.

In the tests, the Length-Driven algorithm was least successful. It was tested for a range of values of $\omega_{L}$. To give a comparison against purely random MCTS, the value zero was included. The results are shown in Table 1. Only above 75% fill did it show any noticeable advantage over purely random filling ($\omega_{L}=0$). At that point, however, the results were already beginning to tail off and 85% fill proved to be the highest level at which it achieved any success. Although the results were disappointing for this algorithm on its own, this test did suggest an optimal value of 0.5 for $\omega_{L}$.

In order to assess the efficacy of the Entropy-Guided Algorithm, the experiments were repeated for different values of the entropy weighting parameter $\omega_{E}$, introduced in Equation (8), Section 5.2. The value of $\omega_{E}$ was varied from 0.96 to 1. This range was chosen because initial experiments had revealed that values very close to, but less than, 1 produced good results. The results of the Entropy-Guided Algorithm are shown in Table 2.

For the Combined Algorithm, the most successful variant of each of the two algorithms were chosen. Thus the values used were $\omega_{L}=0.5$ and $\omega_{E}=0.99$. To combine the two together, a further parameter α was introduced as a linear weighting factor between the two algorithms. The results of this test are shown in Table 3. It can be seen that the best results overall were achieved with α=0.3. However most consistent results for modest fill levels (up to 94%) were achieved by α=0.5; values below α=0.3 appear to work better at the highest levels. In fact, there is little to choose between different values of α in the range 0.1–0.7. The Skyline algorithm worked well for fill levels up to 85%. Above that level, it was outperformed by the Entropy-Guided and Combined algorithms. Above the 85% fill level, the results for the Skyline algorithm were actually evaluated over 250 different sets of stacks in order to give a more statistically accurate result. This was possible because the Skyline algorithm is deterministic and so always gives a very quick result when it works. Results for the Skyline algorithm are compared with the best parameter values of our other algorithms in Table 4. An “overall” column has also been added to the table. This column indicates the number of combinations that are known to be solvable. To obtain this number repeated runs were performed of the most successful algorithm with a much longer time allowance.

### 7.2. Visual Comparisons

**Figure 5:**
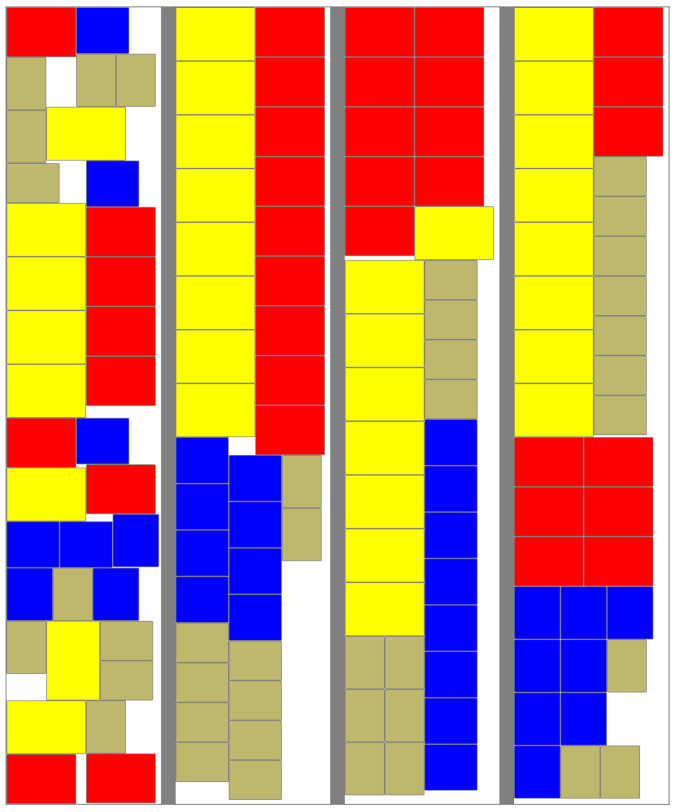
Comparison of layout methods at 85% fill, including (left to right): Length-Driven algorithm with $\omega_{L}=0.5$, Skyline algorithm, Entropy-Driven algorithm with $\omega_{E}=0.99$, Combined algorithm with $\omega_{L}=0.5$, $\omega_{E}=0.99$ and α=0.3.

In a practical application, it is not only a matter of finding an arrangement of stacks that fit, it is also important to find a layout that is practical for the forklift drivers to use. In previous work [7] it was found that low entropy layouts were regarded as preferable by the loaders. Figure 5 shows the layouts created by different algorithm versions for one of the 85% fill sets for which the Length-Driven algorithm was successful. The leftmost image shows the output of that algorithm with $\omega_{L}=0.5$ followed by the Skyline algorithm, the Entropy-Driven algorithm with $\omega_{E}=0.99$ and the Combined algorithm with $\omega_{L}=0.5,\omega_{E}=0.99$ and α=0.3.

**Figure 6:**
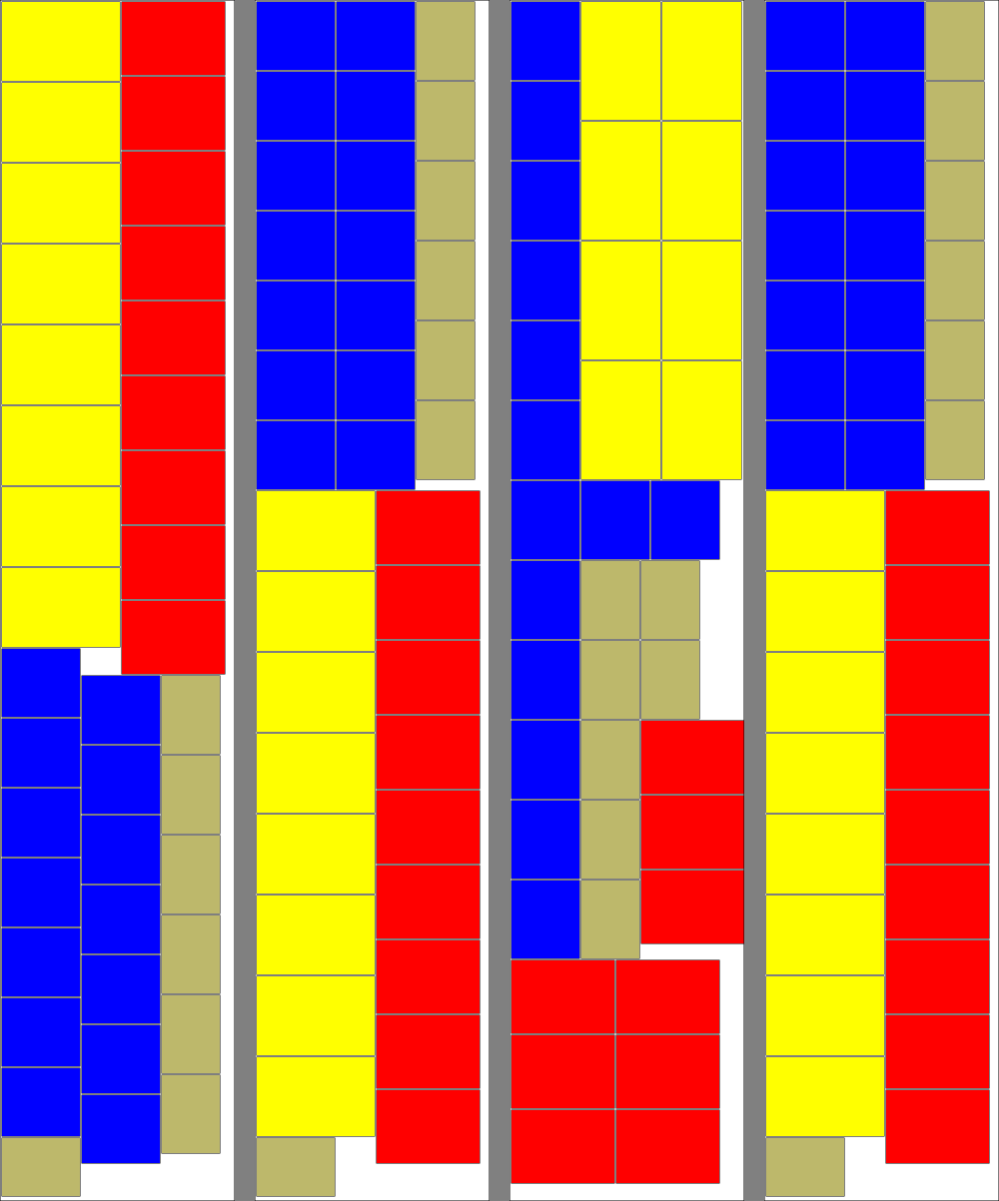
Comparison of Layout Methods at 92% fill where Skyline algorithm still worked, (left to right): Skyline algorithm, Entropy-Driven algorithm with $\omega_{E}=0.99$, Combined algorithm with $\omega_{L}=0.5$, $\omega_{E}=0.99$ with α=0.3 and α=0.7.

**Figure 7:**
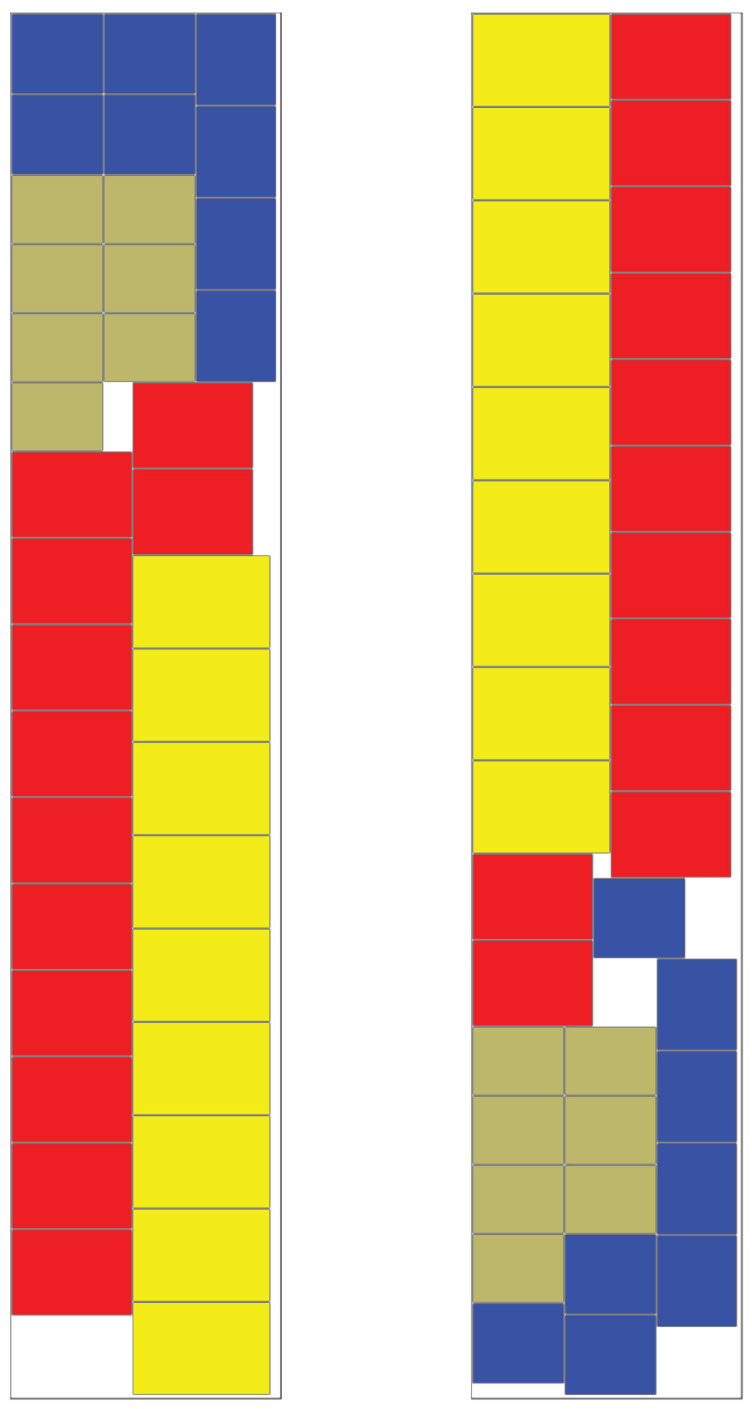
Comparison of Layout Methods at 90% fill where Skyline algorithm failed and only ω=0.96 (left image) and ω=0.97 (right image) succeeded.

It is clear from this image why the length-driven algorithm fails for high fill levels. The relatively unorganised structure of the layout results in embedded empty space that cannot be used for further stacks. At this level of fill, there is little to choose between the remaining algorithms. The highest fill level at which the Skyline algorithm succeeded was 92%. One of these is shown in Figure 6. The order from left to right is: Skyline algorithm, Entropy-Driven algorithm with $\omega_{E}=0.99$, Combined algorithm with $\omega_{L}=0.5$, $\omega_{E}=0.99$ with α=0.3 and α=0.7. The lower values of ω and α do produce less regular patterns but they introduce greater flexibility. As can be deduced from Table 2 and Table 3, this sometimes allows a layout to be found where the variant with a higher value of ω was unsuccessful. In fact, this phenomenon occurs more frequently than the table data implies because of the randomised nature of the data and algorithms. A specific example of this can be seen in Figure 7. This is the case where only ω=0.96 and ω=0.97 were successful. It is notable that the entropy value for ω=0.96 is 29.88 which is lower than the value for ω=0.97, which is 31.84. This is because all versions of the algorithm are searching for the lowest entropy, whereas the value of ω controls the amount of random variation within the search.

## 8. Conclusions and Future Work

In this paper, a new entropy-based approach to solve the problem of generating feasible layouts for the single container loading problem is presented. This approach assists the safe and easy loading of palletised goods by warehouse operatives using forklift trucks. The generality of the approach makes it suitable for dealing with container loading configurations where the types of pallets used are not known beforehand.

While the layouts generated might seem obvious to the reader, this is the very thing that was intended: to have produced a generalised approach implemented on a computer that produces container layouts similar to those produced by expert human operatives with their knowledge of the limitations of the environment. Experts at generating layouts may not always be available and can make mistakes, so an algorithm that can achieve equivalent outcomes in a short time is desirable. Although for simple cases, a similar result can be achieved by simpler methods using the skyline algorithm with an input list sorted by item size, we show that this method is less robust when the fill level is high and often fails where the new technique succeeds.

Moreover, the novel approach of guiding or directing the Monte Carlo Tree Search via the entropy criterion lends itself to generalisation to include further constraints that are pertinent to any real-life situation. Addressing other practical issues such as the weight distribution of goods laterally and longitudinally across the floor of a container, as well as legally enforced axle weight limits can be included in future. A further area to be studied is the generation of “stable” layouts that have very minimal or no lateral motion during container transportation in order to prevent (or reduce) potential damage to goods during transit. Guided Monte Carlo Tree Search provides a means to weight the chosen pallets by multiple criteria, so that the resultant layouts achieve the optimal combination of characteristics. Generating guidance by the inclusion of the expert users’ preferences or importance attached to these criteria will allow this. This inclusion of “soft criteria” is an area that Bortfeldt and Wäscher [5] consider has not been sufficiently explored.

## Figures and Tables

**Table 1:** Overall success for 50 sets at each fill level—Length-Driven algorithm.

Fill Level	$\omega_{L}$ = 0	0.01	0.1	0.3	0.5	0.7	0.9	0.99
0.73	47	49	50	50	50	49	48	49
0.75	43	45	45	48	50	50	48	41
0.77	31	24	34	37	44	40	33	36
0.79	8	9	24	23	33	29	18	18
0.80	0	6	17	25	29	27	18	9
0.81	0	1	1	10	19	11	9	8
0.83	0	4	0	6	10	5	3	2
0.85	0	0	0	0	3	0	1	0
0.87	0	0	0	0	0	0	0	0

**Table 2:** Overall success for 50 sets at each fill level—Entropy-Guided algorithm.

Fill Level	$\omega_{E}$ = 0.96	0.97	0.98	0.99	1.0
0.83	50	50	50	50	50
0.85	48	49	50	49	50
0.87	45	45	49	50	45
0.89	18	31	32	44	28
0.9	12	17	25	36	24
0.91	4	8	10	19	11
0.92	3	3	9	15	6
0.93	2	1	4	3	2
0.94	1	0	1	1	1
0.95	0	0	0	0	1
0.96	0	0	0	0	0

**Table 3:** Overall success for 50 sets at each fill level—Combined algorithm.

Fill Level	α = 0.01	0.1	0.3	0.5	0.7	0.9	0.99
0.87	50	50	50	50	50	50	50
0.89	45	46	46	48	48	46	46
0.90	36	39	43	44	44	44	43
0.91	27	31	27	32	27	25	25
0.92	20	29	25	21	28	23	22
0.93	11	19	27	19	20	15	17
0.94	2	5	7	10	5	4	5
0.95	1	3	2	0	1	1	1
0.96	0	1	0	0	0	0	0

**Table 4:** Overall success for 50 sets at each fill level; comparison of Skyline, Entropy and the Combined algorithm. Note that the “Overall” results show the proportion of the data sets that are known to be solvable after extended runs.

Fill Level	Skyline	Simple Entropy	Combined	Overall
0.85	50	49	50	50
0.87	45	50	50	50
0.89	32	44	46	50
0.90	17	36	43	50
0.91	11	19	27	50
0.92	3	15	25	50
0.93	0	3	27	50
0.94	0	1	7	33
0.95	0	0	2	25
0.96	0	0	0	7
Total	158	217	277	415

## Abbreviations

The following abbreviations are used in this manuscript:

CLP	Container Loading Problem
MCTS	Monte Carlo Tree Search
NMCS	Nested Monte Carlo Search
UKDC	UK Distribution Centre
